# Supplementary material for: An epigenetic switch in vascular phenotype augments anti-tumor immunity
Source: Angiogenesis. 2026 May 19;29(3):33. doi: 10.1007/s10456-026-10042-y (PMC13186828; doi:10.1007/s10456-026-10042-y)
Supplement: Supplementary file 1 — Supplementary file1 (DOCX 1584 KB) [file 10456_2026_10042_MOESM1_ESM.docx]

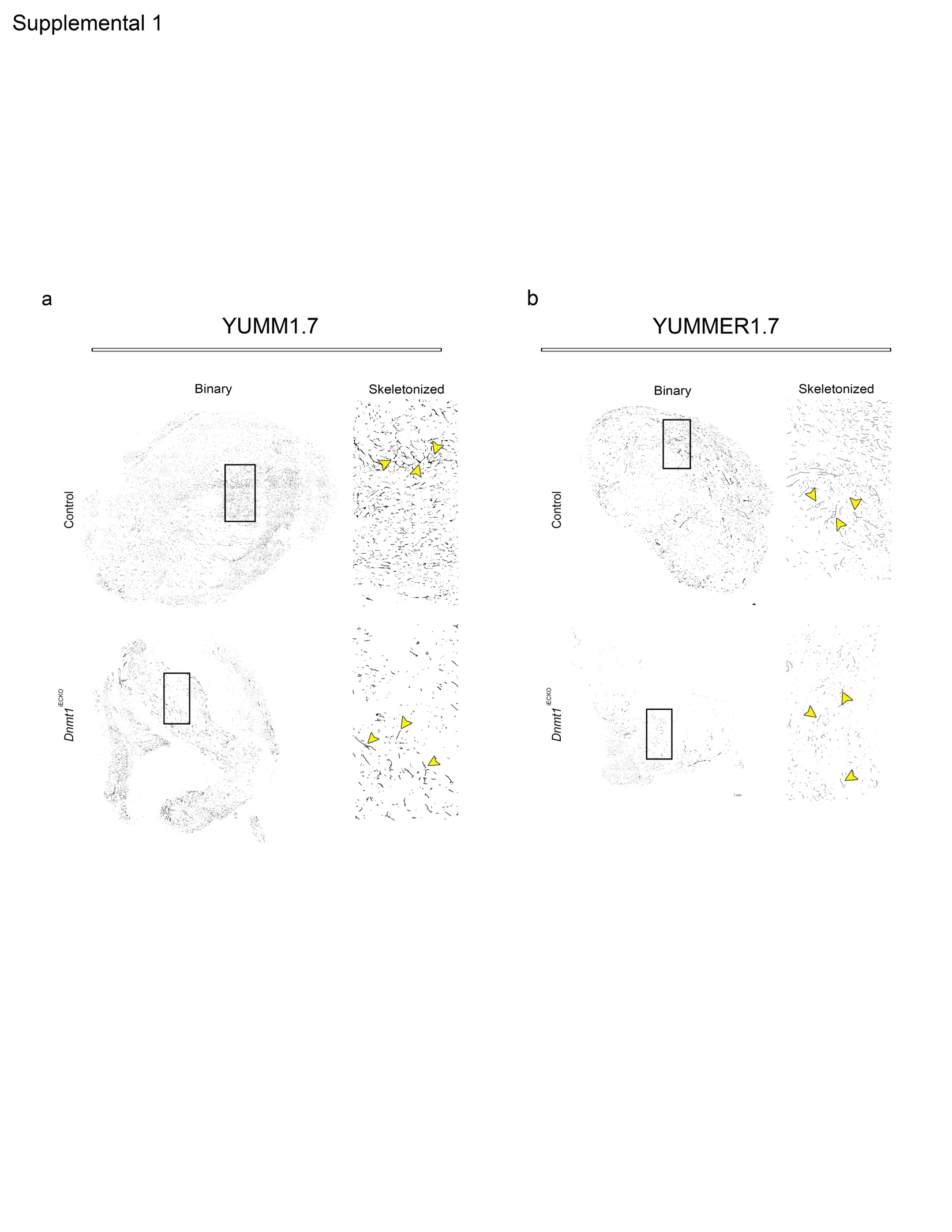


**Supplementary figure 1.** **Blood vessel densities are reduced in both YUMM1.7 and YUMMER1.7 tumors in *Dnmt1*^iECKO^ mice.** (a,b) Representative binary and skeletonized images highlight differences in the density and organization of tumor-associated vasculature. A confocal tile scan captured entire tumors in control and *Dnmt1*^iECKO^ mice. Tumor blood vessels were visualized using ZsGreen labeling and converted into an 8-bit binary image (grey). The magnified boxed areas on the right allow for detailed observation and were inverted to emphasize lateral branches. Notably, arrowheads in control mice indicate branched vessels, contrasting with the straighter, narrower vessels in *Dnmt1*^iECKO^ mice. Scale bars = 1 mm.


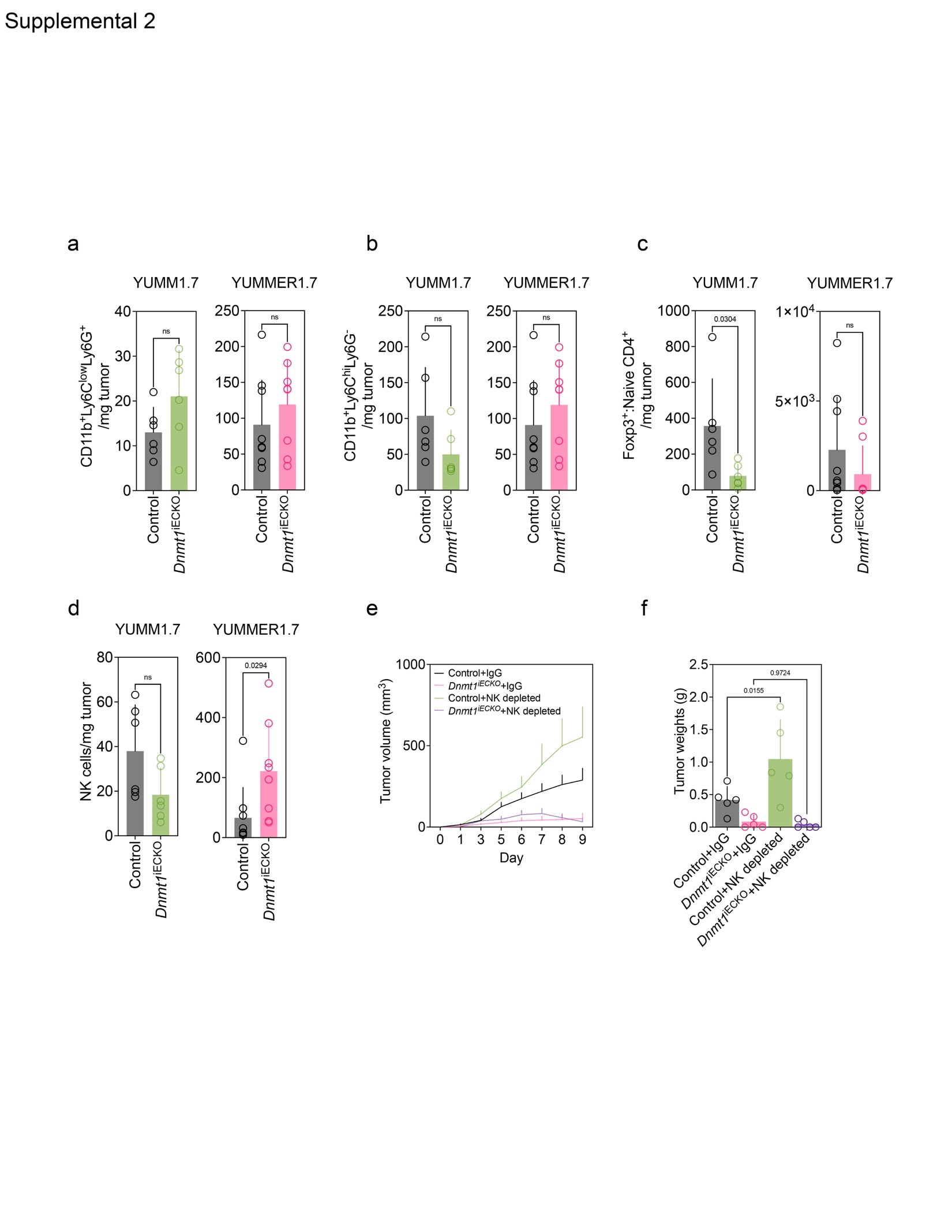


**Supplementary figure 2. NK cells are increased in YUMMER1.7 tumors in *Dnmt1*^iECKO^ mice, but depleting NK cells does not rescue tumor growth.** (a-d) Flow cytometric quantification of myeloid-derived suppressor cells, regulatory T cells, and NK cells in control versus *Dnmt1*^iECKO^ mice. Data analyzed using Student’s *t*-test. YUMMER1.7 growth curves following NK cell depletion. (e) Tumor-bearing mice were treated with anti-NK1.1 or isotype IgG starting from day zero. Tumor volumes were measured daily *(n* = 5 per group). No statistically significant differences were observed between the groups. (f) Tumor weights at endpoint from the same experiment shown in “e”, comparing control and *Dnmt1*^iECKO^ mice treated with IgG or anti-NK1.1 antibodies. For all quantitative analyses in this series, each data point is an individual mouse.


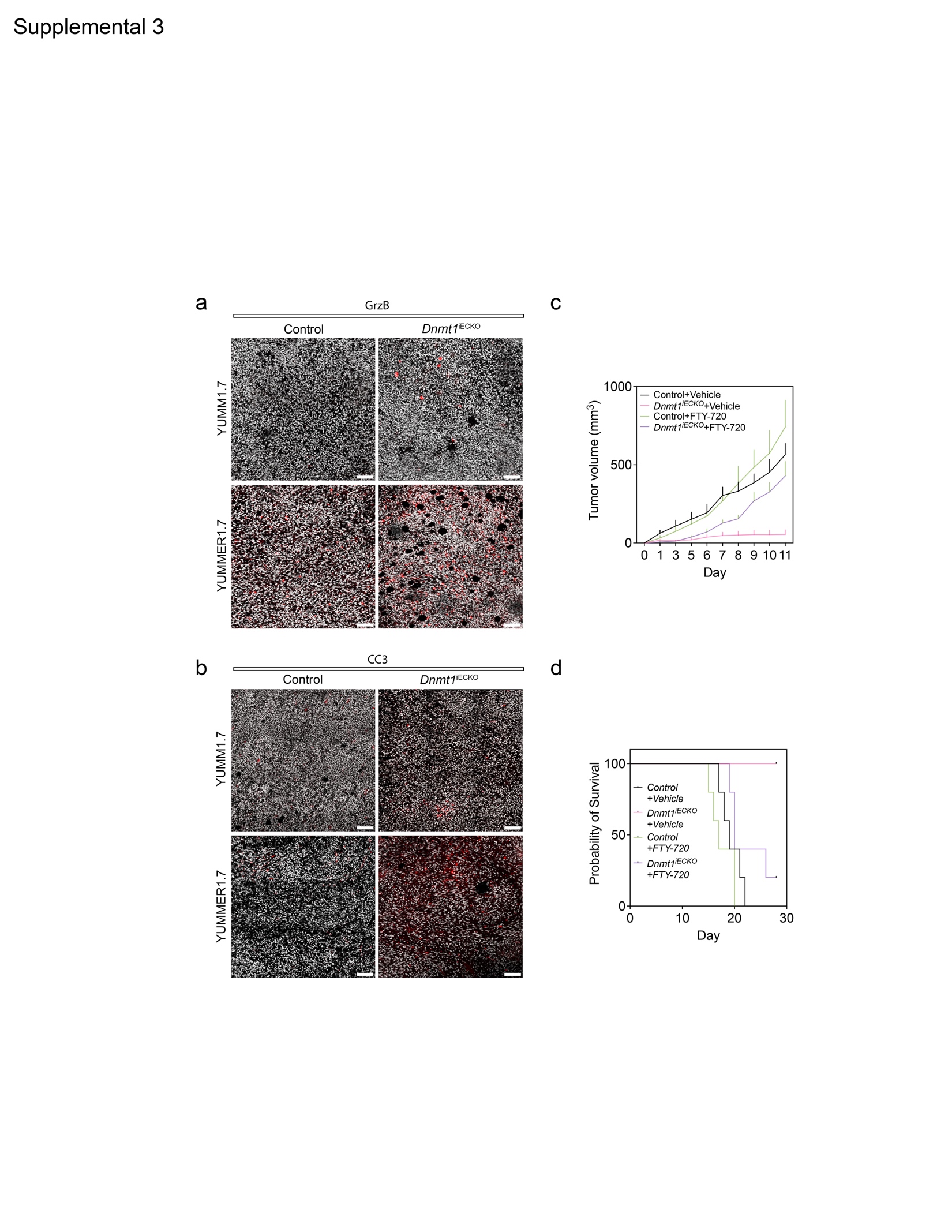


**Supplementary figure 3. GrzB^+^ and cleaved caspase 3 (CC3^+^) cells are increased in YUMMER1.7 tumors in *Dnmt1*^iECKO^ mice.**

(a,b) Representative immunofluorescence images of tumor sections stained for GrzB or CC3 (both shown in red) and counterstained with DAPI (grey). Scale bar = 200 μm. (c,d) Growth and overall survival of control versus *Dnmt1*^iECKO^ mice bearing YUMMER1.7 tumors and treated with FTY720.


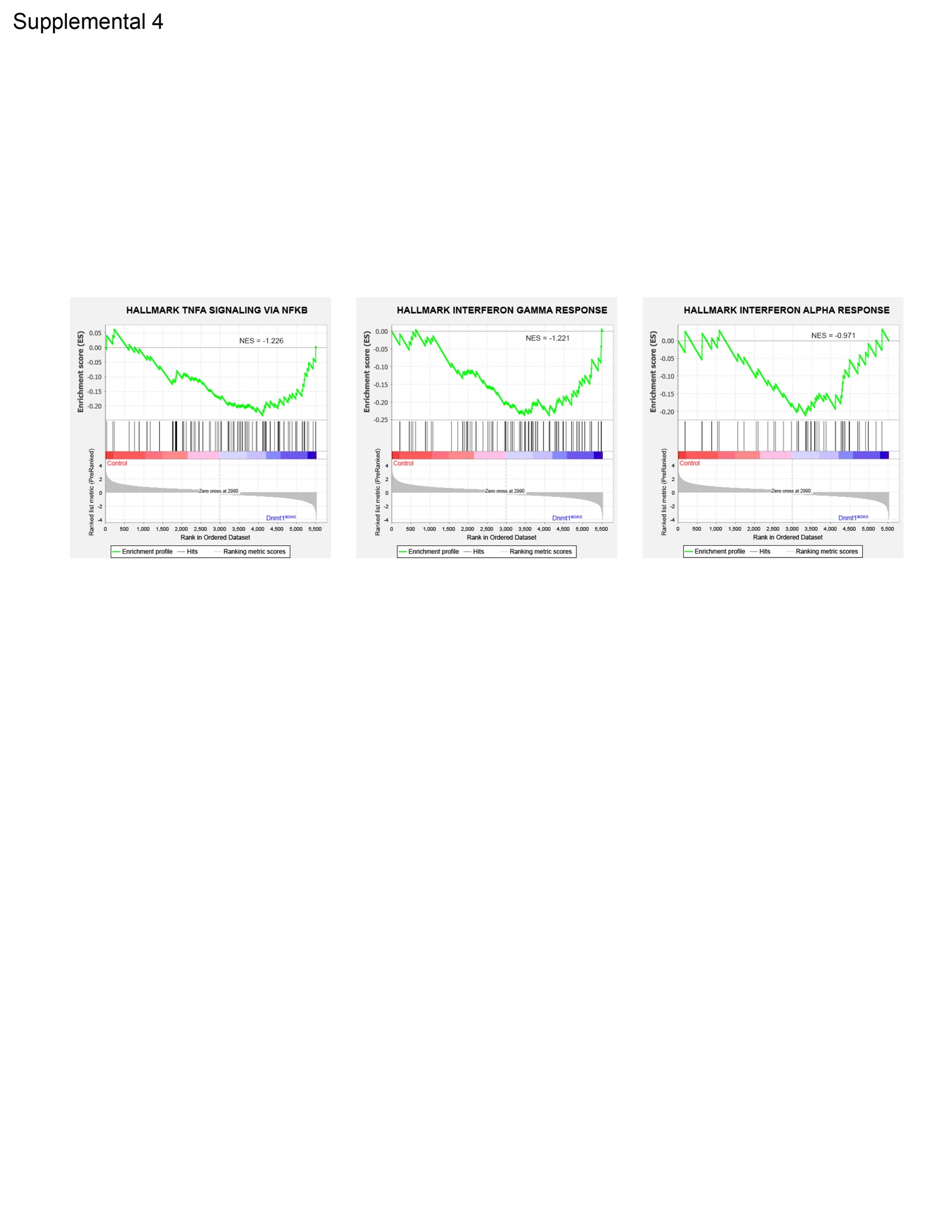


**Supplementary figure 4. Enrichment scores in control versus *Dnmt1*^iECKO^ TECs.** The plots showing trending ES for Hallmark TNFα, IFNγ, and IFNα response genes.
